# Quality of Life and Needs Assessment of Cancer Patients’ Caregivers in the Saudi Culture

**DOI:** 10.7759/cureus.27591

**Published:** 2022-08-02

**Authors:** Hani S Almugti, Maram M Shaiea, Yazeed M Alhammadi, Razan A Mawkili, Shereen Alemam, Rawabi A Hamas, Abdulrahman M Jurebi, Siraj B Alharthi, Feras I Al-Hazmi, Mohai E Bali, Haitham F Asiri, Meshal A Al Jutayli, Farees Y Almohaimeed, Eman M Owaisheer, Abdul-Qader M Alaa Adeen

**Affiliations:** 1 Ministry of National Guard Health Affairs, King Abdullah International Medical Research Center, King Saud bin Abdul-Aziz University for Health Sciences, Jeddah, SAU; 2 Pharmacy, Jazan University, Jazan, SAU; 3 College of Medicine, King Faisal University, Al-Ahsa, SAU; 4 College of Medicine, Jazan University, Jazan, SAU; 5 Respiratory Therapy, King Abdullah Medical City Specialist Hospital, Makkah, SAU; 6 Infection Control, King Fahad Hospital, Tabuk, SAU; 7 College of Medicine, Batterjee Medical College, Jeddah, SAU; 8 Biological Sciences, King Abdul-Aziz University, Taif, SAU; 9 Adult Intensive Care Unit, King Fahad Specialist Hospital, Dammam, SAU; 10 Dental Public Health, Ministry of Health Holdings, Asir, SAU; 11 Pharmacy, Hayat National Hospital, Qassim, SAU; 12 College of Medicine, Qassim University, Qassim, SAU; 13 Radiology, Maternal and Child Hospital, Hafar Al Batin, SAU; 14 College of Medicine, King Saud bin Abdul-Aziz University for Health Sciences, Makkah, SAU

**Keywords:** saudi culture, needs, quality of life, caregivers, cancer

## Abstract

Background: Cancer is a chronic health condition that requires long-term treatment and care. Diagnosis of cancer is a family crisis that has a bad impact on patients and their caregivers, which can worsen the quality of life of the entire family members. It would be relevant to highlight the changes in the quality of life among cancer patients' caregivers within the Saudi culture to strengthen their involvement in the plan of treating cancer patients.

Objective: To assess the needs and quality of life of Saudi cancer patients' caregivers by using the World Health Organization quality of life questionnaire WHOQOL-BREF and the Family Inventory of Needs (FIN) questionnaire for family caregivers.

Materials and methods: Of 376 caregivers invited to participate, 270 (72%) accepted and completed the questionnaire. The study was carried out in outpatient clinics and oncology inpatient wards of Princess Noorah Oncology Center in King Abdul-Aziz Medical City Jeddah, Saudi Arabia.

Results: The study found that 53 % of Saudi caregivers reported a good quality of life in the following domains: psychological, social relationship, and environment. On the other hand, the lower quality of life scores were stated for the physical health domain in almost two third of participants (67 %). Poor quality of life was reported among the male caregivers of the older age group who had a lower level of education and had a short term of caring (fewer than 12 months). Regarding the need assessment, most of the 20 needs items were rated important and were related to patient care. Whereas, the least important needs were related to the caregivers' health.

Conclusion:There was a significant association between quality of life scores and the demographic characteristics of the caregivers, addressing these factors in addition to the assessment of the caregivers' needs during medical care will provide holistically care for the patients and their caregivers to increase their quality of life.

## Introduction

Cancer is a major public health problem globally, responsible for 10 million deaths in 2020 [1]. Over the past years, most researchers have predicted an increase in cancer cases due to population aging and growth [2]. Consequently, there will be more psychological and social-economical burdens on the patients, their families, friends, and the health care services [3].

Between 1990 and 2016, cancer incidence in Saudi Arabia increased up to 10-fold for breast and colon cancer and 8-fold for prostate cancer [4]. A large volume of published studies describes the role of an unhealthy lifestyle as an attributed factor to cancer [5,6]. Compared to the developed countries, Saudi Arabia is considered one of the developing countries facing an increased incidence of cancer due to their populations' adoption of western lifestyles [6].

A cancer diagnosis is a pivotal event in the patients' lives and the lives of their families. Previous studies have reported psychological and physical distress experienced by cancer patients [3,7], and other studies found a higher impact on family members than on patients [8]. These negative impacts last for many years if we consider cancer a chronic health condition that needs continuous care as more than half of cancer patients have a high survival rate.

Cancer patients have multiple care needs, including health counseling, effective treatment options, symptoms management, controlling the side effects of treatment, emotional support, assistance with personal care, and follow-up appointments with the health care provider [9]. Family caregivers used to take an essential role in most of the patient's needs, thus urging health care providers to involve them in the medical management plan [8,9].

Concerning the negative consequences of caring for a person with cancer, in addition to psychological distress and restriction of social activities, the previous studies reported sleep disturbance, weight loss, fatigue, reduced physical activity, and loss of appetite [7]. Further, one study found that at least two years of cancer patient care is associated with clinical depression for 52.9% of the caregivers [10]. Indirectly, these burdens reduce the well-being of cancer patients who need all support.

Lastly, the caregivers play a crucial role in the cancer management plan [3], have an emotional response to the patients' diagnosis and prognosis, and may require coaching and emotional support [11]. Families with different cultures have specific communication and coping styles [12]. In Saudi culture, there is limited research about the quality of life among cancer patients' caregivers. In order to strengthen the caregivers' role in the management plan, it is important to assess their needs to help stakeholders initiate an effective support program for them.

## Materials and methods

Aim of the study

Improve the provided health care to Saudi cancer patients by highlighting the changes in the quality of life among cancer patients' caregivers.

Primary (Specific) Objectives

(1) To assess the quality of life among Saudi cancer patients' caregivers using the World Health Organization quality of life questionnaire WHOQOL-BREF. (2) To assess the needs of Saudi cancer patients' caregivers using the Family Inventory of Needs (FIN) questionnaire for family caregivers.

Study area/setting

The study was carried out in outpatient clinics and oncology inpatient wards of Princess Noorah Oncology Center in King Abdul-Aziz Medical City Jeddah, Saudi Arabia.

Study Subjects

The study recruited cancer patients' caregivers who had the following inclusion criteria: Saudi nationality, age more than 18 years, and direct close and supportive caregiver. The caregivers of a patient in palliative care were excluded from our study.

Study Design and Sample Size

This is a cross-sectional study. The target sample size is 376 Saudi cancer patients' caregivers at a 5% margin of error and confidence level of 95% (assuming there is only one caregiver for each cancer patient in Princess Noorah Oncology Center, and the annual number of cancer cases is 17,602 in all oncology centers in Saudi Arabia [13]).

Sampling Technique

Through a convenient sampling technique (non-probability sampling), the study recruited cancer patients' caregivers who met the inclusion and exclusion criteria using a self-administered questionnaire.

Data collection methods, instruments used, measurements

Variables

The following variables were considered in this study - (a) Dependent variables: quality of life and the needs of cancer patients' caregivers; (b) Independent variables: age of caregivers, gender of caregivers, level of education, relation to the patient, Living situation, duration of giving care to patients, type of cancer, stage of patient cancer.

Quality of Life

Quality of life [14] is defined as the self-assessment of the individual's life position in relation to the values, goals, standards, and concerns shared with the culture. World Health Organization quality of life questionnaire WHOQOL-BREF expands this definition to be a multi-dimensional structure involving health status, lifestyle, life satisfaction, mental state or well-being.

Needs of Cancer Patients' Caregivers

It is essential to address caregivers' met and unmet needs as they have a positive intervention in caring for cancer patients. Family Inventory of Needs [15] is recognized as the measurement tool that identifies the needs in two subscales. The first one measures the importance of 20 care needs, and the second subscale measures whether those important needs have been met, partly met or not.

Questionnaire

This study utilized a self-administered type of questionnaire (included in the appendix) that consisted of three sections as the following: (a) the first section includes the demographic data; (b) the second section includes the assessment of the quality of life using the World Health Organization quality of life questionnaire WHOQOL-BREF; the validity and reliability of this measurement tool are ensured by the WHOQOL Group, Programme on Mental Health [14]. There are 19 different languages of WHOQOL-BREF, and we used the Arabic version. The WHOQOL-BREF contains four domains (Physical health, Psychological, Social Relationships, and Environment) that score by 26 questions. Good quality of life is granted if the score value is more than the mean value; and (c) the third section includes a needs assessment of Saudi cancer patients' caregivers using the Family Inventory of Needs (FIN) questionnaire [15]. In order to validate the questionnaire, it was translated to Arabic and then to English and revised by a professional team of specialists in family medicine, oncology, community medicine, and mental health. Additionally, the questionnaire's internal consistency was examined for reliability, and the results showed high reliability with a Cronbach alpha rating of 0.8. The family Inventory of Needs (FIN) questionnaire contains 20 items, each of which is rated on two subscales. The first subscale measures the importance of 20 care needs (the response options range from 1 (not important) to 5 (very important). The second subscale measures whether those needs rated as important have been met, partly met, or not.

Study plan

Over a two-month period, the data collection of the present study was conducted following the approval of the study (NRJ22J/071/03) from the King Abdullah International Medical Research Center. Throughout the course of the study, we rigorously protected the privacy of the participant's information, and we got informed consent from the participants prior to the data collection process.

Data analysis

For data entry and analysis, the SPSS statistical software package for Windows was used (version 20.0; IBM Corp., Armonk, NY, USA). Quality control was maintained at the coding and data entry steps. Data are presented using descriptive statistics in the form of frequencies and percentages for qualitative variables and means and standard deviations for quantitative variables. The Chi-square test and Fisher exact test were used to test the association between participants' answers and their demographic characteristics.

## Results

Characteristics of the study subjects

**Figure 1:**
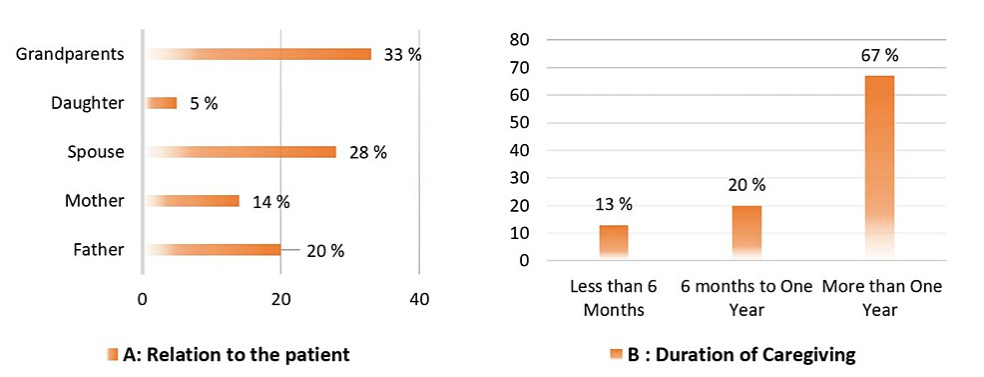
Percentages of participants according to their relationship with the patient (A) and their duration of caregiving (B)

The response rate was 72% of 378 caregivers who were invited, 270 agreed to participate and completed the study. Table 1 shows the general characteristics of participants and their patients. The mean age of participants was 26 ± 7 years, ranging from 19-40 years. The cancer patients had a mean age of 54 ± 15 years, and more than half of them were males who had been diagnosed with colon cancer for more than one year. Almost two-thirds of the caregivers were female and lived with the patients in the same house. Most caregivers (80%) have at least a Bachelor's degree, and almost two-thirds spend more than one year on direct caregiving (Figure 1).

**Table 1:** Demographic characteristics of participants (n=270) * Standard Deviation

Demographic characteristics	Frequency	Percent (%)
Caregivers' characteristics		
Age		
Range	19- 40 years	
Mean ± SD*	26 ± 7 years	
Gender		
Male	95	33
Female	190	67
Level of Education		
High school	54	20
Bachelor degree	162	60
Master/PHD	54	20
Living Situation		
Same household	180	67
Different household	90	33
Patients' characteristics		
Age		
Range	22- 72 years	
Mean ± SD*	54 ± 15 years	
Gender		
Male	183	68
Female	87	32
Type of Cancer		
Colon	152	56
Breast	19	7
Lymphoma	76	28
Others: Leukemia - Stomach	23	9

Quality of life assessment

**Figure 2:**
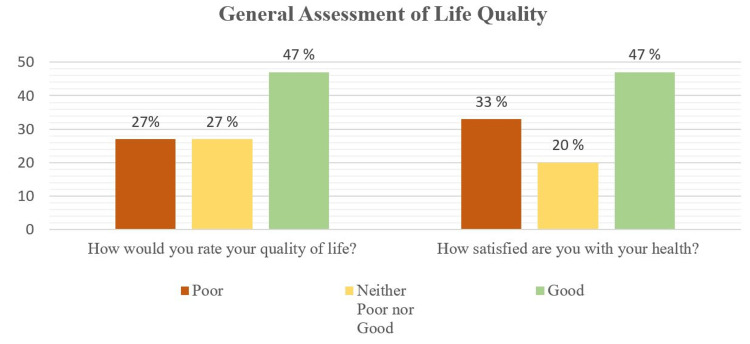
Percentages of participants' answers to the first two general questions of WHOQOL-BREF (n=270)

Concerning the quality of life assessment, Figure 2 illustrates the participants' answers to the first two general questions of WHOQOL-BREF; almost half of the participants reported good quality of life and a good rate of health satisfaction.

Table 2 shows that although two-thirds of participants (67 %) had a lower score of quality of life in the physical health domain, mean total scores indicate good quality of life among half of all participants in the present study.

**Table 2:** Participants' mean score and the rate of the quality of life dimensions (n=270) * Good quality of life is indicated if the score ≥ means value.

Quality of Life Domains	Mean score	Rate of Good*	Standard Deviation	Minimum- Maximum score value
Physical health	47	33 %	10	37 - 69
Psychological	50	53 %	22	19 - 94
Social relationships	47	53 %	18	25 - 75
Environment	52	53 %	17	25 - 75
Whole Domains	49	48 %	-	-

Important family care needs and unmet needs

Most of the 20 needs assessed with FIN were considered important. The mean score of the important needs was 3.6 (Table 3). 70 % of the participants rated the needs related to patient care as important. In contrast, the least important needs were related to the caregivers' health. The highest three unmet needs that were reported and rated as important by participants: feel that the health professionals care about the patient (unmet by 45% of the participants); having information about what to do for the patient at home (unmet by 43% of the participants); and help with the patient's care (unmet by 49% of the participants).

**Table 3:** Family care needs measured by FIN (n=270) * Needs scale: 1 (not important) – 5 (very important) ** Standard Deviation *** Prevalence of important needs scored 4 (important) or 5 (very important) FIN: Family Inventory of Needs questionnaire

FIN-Family Care Needs	Mean* ± SD**	Important needs % ***	Unmet needs %
Have my questions answered honestly?	4.2 ± 0.9	80	7
Know specific facts concerning the patient's prognosis	4.4 ± 1	40	20
Feel that the health professionals care about the patient	4 ± 1	73	45
Be informed of changes in the patient's condition	4 ± 1.4	67	13
Know exactly what is being done for the patient	3.8 ± 1.1	60	20
Know what treatment the patient is receiving	3 ± 0.8	73	7
Have explanations given in terms that are understandable	3.67 ± 0. 9	60	27
Be told about treatment plans while they are being made	4 ± 1	67	7
Feel there is hope	3.9 ± 1	53	13
Be assured the best possible care is being given to the patient	4 ± 1.7	60	10
Know what symptoms the treatment or disease can cause	3.9 ± 1	67	30
Know when to expect symptoms to occur	3 ± 1.2	33	7
Know the probable outcome of the patient's illness	3.3 ± 1.2	80	20
Know why things are being done for the patient	4.2 ± 0.75	47	22
Know the names of health professionals involved in the patient's care	3.7 ± 0.6	60	40
Have information about what to do for the patient at home	3.9 ± 0.6	83	43
Feel accepted by the health professionals	4 ± 0.9	60	13
Help with the patient's care	3.8 ± 0.8	79	49
Have someone be concerned with my health	3.8 ± 0.7	60	20
Be told about people who could help with problems	4 ± 0.8	73	7

Relation between participants' quality of life domains and their demographic characteristics

Table 4 demonstrates the statistical significance (p < 0.05) between the different quality of life domains and most participants' characteristics. The mean scores of quality of life domains were significantly higher among the participants who were younger, female, and had a high level of education. Moreover, the participants living together with their patients had a good quality of life compared to participants living in different households. Participants reported good quality of life if they had experience in caregiving for more than one year. Although there is no statistical significance between the quality of life and type of relation to the patients, a higher quality of life score was reported for the participant who took care of grandparents.

**Table 4:** Relation between participants' quality of life domains and their demographic characteristics (n=270) (*) Statistically significant at p < 0.05

Demographic characteristics	Quality of Life Domains										
	Physical health			Psychological		Social relationships		Environment			
	Poor		Good	Poor	Good	Poor	Good	Poor	Good		
Age group											
18 – 25 Years	36		126	72	90	45	108	80	82		
26 – 35 Years	36		36	36	36	13	59	23	49		
> 36 Years	18		18	20	16	23	19	20	16		
P value	< 0.001*			< 0.001*		< 0.001*		< 0.001*			
Gender											
Male	54		36	54	36	18	72	18	72		
Female	36		144	54	126	72	108	54	126		
P value	< 0.001*			< 0.001*		< 0.001*		0.003*			
Level of Education											
High school	36		18	34	20	28	26	38	16		
Bachelor degree	36		126	90	72	72	90	72	90		
Master/PHD	36		18	18	36	0	54	0	54		
P value	< 0.001*			< 0.001*		< 0.001*		< 0.001*			
Relation to the Patient											
Father	0		53	0	53	0	53	0	53		
Mother	0		38	29	9	19	19	29	9		
Spouse	51		25	25	51	0	76	0	76		
Daughter	6		7	6	7	13	0	7	6		
Grandparents	36		54	18	72	18	72	0	90		
P value	0.079			0.76		0.58		0.071			
Living Situation											
Same household	36		144	90	90	72	108	54	126		
Different household	54		36	18	72	18	72	18	72		
P value	< 0.001*			< 0.001*		< 0.001*		< 0.001*			
Duration of caregiving											
Less than 6 months	0	36		18	18	36	0	18	18		
From 6 months to One Year	18	36		28	26	18	36	36	18		
More than One Year	72	108		36	144	36	144	18	162		
P value	< 0.001*			< 0.001*		< 0.001*		< 0.001*			

## Discussion

The present study was designed to assess the caregivers' quality of life. The results indicate that two-thirds of the participants were females who lived with their patients in the same house; this aligns with findings observed in earlier studies and recognizes females as the primary caregivers in their family [16]. Further, It is interesting to note that two-thirds of cancer patients were male; this supports the data from the World Health Organization (2018), which states that cancer incidence among males is higher than among females. Regarding the relationship with patients, the current study found that almost a third of participants were patients' spouses or partners, consistent with previous studies and revealing the high quality of the relationship between spouses within the Saudi Muslim culture [16,17].

The present sample's overall quality of life score was considered good, and three out of four domains had mean scores above 50. This finding is consistent with another study conducted in Saudi Arabia, although the data collection tool was not similar. On the contrary, this result is considered high compared to other previous studies; however, it explains the Saudis' ability to cope when a family member is diagnosed with cancer [16].

The present study demonstrates that the participants' good or poor quality of life is associated with their demographic characteristics. For instance, older caregivers tended to have a poor quality of life compared with younger caregivers. This might occur because older people have physiological, psychological, and social changes that worsen their quality of life, indirectly affecting the quality of care for their patients. Moreover, the female caregivers reported good quality of life scores, thus supporting the cultural characteristics of Saudi Arabia, where women can adapt to their families' crises and have a leading role as a nurse in caring for sick family members. In contrast, the men have a role as the family's backbone, with difficulty splitting their time between working and caring for their patients, making their quality of life scores lower than females.

Concerning the caregivers' level of education, the present study revealed that caregivers with only a high school degree reported poor quality of life. This is consistent with those who consider that caregivers with low education have lower awareness and poor perception of the health condition of cancer, which could lower their quality of life [18]. However, this finding differs from the previous study that reported poor quality of life among higher educated caregivers who have a high risk of stress due to involving themselves in medical decisions [19]. The findings also showed that caregivers who cared for their patients for less than six months or 12 months had poor quality of life compared to those who had cared for more than 12 months. This finding explains the high demand for care and the burdens at the time of initial diagnosis.

The needs assessment of the family caregiver is the practical approach to improving the family's health outcomes. In this study, the majority of the 20 assessed needs were considered important. The most important needs were related to patient care and the least important needs belonged to the caregivers' health. This has been shown by a previous study [20], highlighting that the caregivers focus on their patients' needs rather than their needs.

There are some limitations of this study. First, as the study was cross-sectional, there are no inferences about causality can be drawn. Second, there was a limited ability to generalize the finding as the number of participants was small, and the study was conducted in one oncology canter. Nevertheless, the current study had several advantages over previous similar research. One advantage was using a recognized, valid, and reliable data collection tool. Also, to the best of our knowledge, this is the first Saudi study aimed at assessing the quality of life and needs assessment together.

## Conclusions

The cancer patients' caregivers in Saudi culture are family members who have the characteristics of providing long-term care to their relatives. The results of the present study show that the caregivers of patients with cancer are prone to have a good quality of life that is influenced positively by being from the female gender, young, and having a high education level. The other significant finding to emerge from this study is that caregivers' needs are rated as important from their perspective. These needs should be addressed at all times during medical care to improve health care. 

## Appendices

The questionnaire consisted of three sections.

(a) The first section includes the demographic data as the following: Caregivers' characteristics: Age of caregivers, gender of caregivers, level of education, relation to the patient, Living situation,  duration of giving care to patients,  Number of cancer patients in the family; Patient's characteristics: Age, gender, type of cancer, stage of cancer.

(b) The second section for the assessment of the quality of life. The first two questions are general and do not represent any domain used to be recorded separately from the remaining 24 questions: Question one: asks about an individual's overall perception of quality of life; Question two: asks about an individual's overall perception of their health.

Physical health domain (7 questions)

1)     To what extent do you feel that physical pain prevents you from doing what you need to do?

2)     How much do you need any medical treatment to function in your daily life?

3)     Do you have enough energy for everyday life?

4)     How well are you able to get around?

5)     How satisfied are you with your sleep?

6)     How satisfied are you with your ability to perform your daily living activities?

7)     How satisfied are you with your work capacity?

Psychological domain ( 6 questions)

1)     How much do you enjoy life?

2)     To what extent do you feel your life to be meaningful?

3)     How well are you able to concentrate?

4)     Are you able to accept your bodily appearance?

5)     How satisfied are you with yourself?

6)     How often do you have negative feelings such as blue mood, despair, anxiety, and depression?

Social relationships domain (3 questions)

1)     How satisfied are you with your relationships?

2)     How satisfied are you with your sex life?

3)     How satisfied are you with the support you get from your friends?

Environment domain ( 8 questions)

1)     How safe do you feel in your daily life?

2)     How healthy is your physical environment?

3)     Have you enough money to meet your needs?

4)     How available is the information you need in your day-to-day life?

5)     To what extent do you have the opportunity for leisure activities?

6)     How satisfied are you with the conditions of your living place?

7)     How satisfied are you with your access to health services?

8)     How satisfied are you with your transport?

(c) The third section for the needs assessment and consisted of the following items:

1)     Have my questions answered honestly?

2)     Know specific facts concerning the patient's prognosis?

3)     Feel that the health professionals care about the patient?

4)     Be informed of changes in the patient's condition?

5)     Know exactly what is being done for the patient ?

6)     Know what treatment the patient is receiving?

7)     Have explanations given in terms that are understandable ?

8)     Be told about treatment plans while they are being made?

9)     Feel there is hope?

10)  Be assured the best possible care is being given to the patient?

11)  Know what symptoms the treatment or disease can cause?

12)  Know when to expect symptoms to occur?

13)  Know the probable outcome of the patient's illness?

14)  Know why things are being done for the patient?

15)  Know the names of health professionals involved in the patient's care?

16)  Have information about what to do for the patient at home?

17)  Feel accepted by the health professionals?

18)  Help with the patient's care

19)  Have someone be concerned with my health

20) Be told about people who could help with problems
